# Neural Signals in Red Nucleus during Reactive and Proactive Adjustments in Behavior

**DOI:** 10.1523/JNEUROSCI.2775-19.2020

**Published:** 2020-06-10

**Authors:** Adam T. Brockett, Nicholas W. Hricz, Stephen S. Tennyson, Daniel W. Bryden, Matthew R. Roesch

**Affiliations:** ^1^Department of Psychology, University of Maryland, College Park, Maryland 20742; ^2^Program in Neuroscience and Cognitive Science, University of Maryland, College Park, Maryland 20742

**Keywords:** cognitive control, inhibition, motor, red nucleus, single-neuron recording, stop signal

## Abstract

The ability to adjust behavior is an essential component of cognitive control. Much is known about frontal and striatal processes that support cognitive control, but few studies have investigated how motor signals change during reactive and proactive adjustments in motor output. To address this, we characterized neural signals in red nucleus (RN), a brain region linked to motor control, as male and female rats performed a novel variant of the stop-signal task. We found that activity in RN represented the direction of movement and was strongly correlated with movement speed. Additionally, we found that directional movement signals were amplified on STOP trials before completion of the response and that the strength of RN signals was modulated when rats exhibited cognitive control. These results provide the first evidence that neural signals in RN integrate cognitive control signals to reshape motor outcomes reactively within trials and proactivity across them.

**SIGNIFICANCE STATEMENT** Healthy human behavior requires the suppression or inhibition of errant or maladaptive motor responses, often called cognitive control. While much is known about how frontal brain regions facilitate cognitive control, less is known about how motor regions respond to rapid and unexpected changes in action selection. To address this, we recorded from neurons in the red nucleus, a motor region thought to be important for initiating movement in rats performing a cognitive control task. We show that red nucleus tracks motor plans and that selectivity was modulated on trials that required shifting from one motor response to another. Collectively, these findings suggest that red nucleus contributes to modulating motor behavior during cognitive control.

## Introduction

An important aspect of human cognition is the ability to inhibit automatic behaviors and to exert control to drive behavior appropriately. Deficits in these abilities are hallmark symptoms of numerous neuropsychiatric disorders (Dalley and Robbins, 2017). Across species and in clinical populations, a common paradigm used to examine both response inhibition and cognitive control is the stop-signal task (Verbruggen and Logan, 2008; Eagle and Baunez, 2010; Verbruggen et al., 2019). During performance of the stop-signal task, participants are required to inhibit (i.e., STOP) an automatic response (i.e., GO response) on a low percentage of trials (typically, ∼20–30%; Verbruggen et al., 2019). Cognitive control is assessed by how well participants inhibit behavior on STOP trials as well as how participants adjust their behavior after difficult or errant trials (i.e., slow responding to increase accuracy; conflict adaptation).

Numerous studies have used variants of the stop-signal task to elucidate the neural signals that give rise to accurate stop-signal performance focusing primarily on “control signals” from frontal regions such as anterior cingulate cortex and medial prefrontal cortex (Pardo et al., 1990; Kolling et al., 2012; Shenhav et al., 2014; Bryden and Roesch, 2015; Bryden et al., 2016, 2019; Tennyson et al., 2018), and “action selection signals” from the basal ganglia (Frank, 2006; Bryden et al., 2012; Schmidt et al., 2013; Wiecki and Frank, 2013; Mallet et al., 2016; Wessel and Aron, 2017). However, little is known about how motor pathways tasked with the planning and implementation of motor responses respond to/adapt their outputs to control signals. Understanding how motor signals are adapted in response to top down modulation is critical, as correct “cognitive computations” are meaningless without accurate and well timed motor commands.

To begin to address this, we characterized neural firing in the red nucleus (RN), a key node in the descending motor pathway (Massion, 1967). The RN receives projections from the interpositus and dentate nuclei of the cerebellum, as well as from the premotor cortex (Onodera, 1984; Houk, 1991; Keifer and Houk, 1994; Onodera and Hicks, 2009). Projections to RN are distinct with axons from the interpositus nucleus targeting the magnocellular division of RN (Houk, 1991; Keifer and Houk, 1994; Onodera and Hicks, 2009), and premotor projections, as well as dentate nucleus projections, targeting the parvocellular division (Murray and Gurule, 1979; Huisman et al., 1981; Onodera, 1984; Onodera and Hicks, 2009; Beitzel et al., 2017). While the importance of these cellular divisions is debated (Houk, 1991; Onodera and Hicks, 2009; Gruber and Gould, 2010), the overall output of RN has been strongly linked to the control of goal-directed movements (Ghez and Kubota, 1977; Burton and Onoda, 1978; Amalric et al., 1983; Cheney et al., 1988; Martin and Ghez, 1988; Dormont et al., 1989; Jarratt and Hyland, 1999; Belhaj-Saïf and Cheney, 2000; van Kan and McCurdy, 2001; Van Kan and McCurdy, 2002; Pacheco-Calderón et al., 2012; Herter et al., 2015).

While much work has focused on the role of RN in motor control, to our knowledge, no study has examined firing in the context of cognitive function. Single-unit studies in cats, rodents, and primates have demonstrated enhanced firing during motor adjustments to physical perturbations in gait, arm position, and grasping movements (Ghez and Kubota, 1977; Cheney et al., 1988; Jarratt and Hyland, 1999; Muir and Whishaw, 2000; van Kan and McCurdy, 2001; Van Kan and McCurdy, 2002; Herter et al., 2015). We hypothesized that RN in rats might serve a similar role when rapid adjustments in behavior are required, as well as when trial-to-trial adjustments occur based on past experience (i.e., conflict adaptation). To test this hypothesis, we recorded from RN in rats performing a novel variant of the stop-signal task. We found that RN showed amplified directional signals on STOP trials and that firing was strongly correlated with accuracy and movement speed, and that modulation of firing in RN reflected trial-to-trial adjustments in cognitive control.

## Materials and Methods

### 

#### 

##### Animals.

Four male and three female Long–Evans rats (*n* = 7; weight at arrival, 175–200 g) were obtained from Charles River Laboratories. Rats were housed on a 12 h light/dark schedule with lights on at 6:00 A.M. Eastern Standard Time (EST). All training, behavioral testing, and recordings occurred between 9:00 A.M. and 2:00 P.M. EST. This study was approved by the Institutional Animal Care and Use Committee and conformed to the National Research Council guidelines (National Research Council (US) Committee for the Update of the Guide for the Care and Use of Laboratory Animals, 2011).

##### Stop-change task.

**Figure 1.**
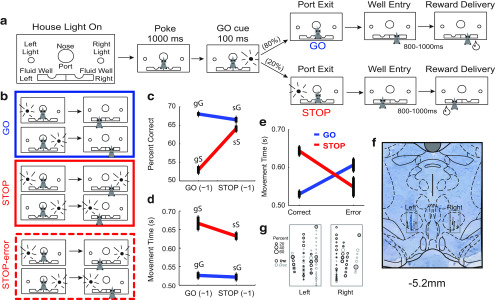
Task design and behavioral analysis. ***a***, Schematic of stop-change task. Following the house lights, rats made a nose poke for 1000 ms before a light cue was illuminated on either the right or left side. In 80% of trials (GO trials), this light corresponded to the correct direction that the rat needed to move to receive the reward. On 20% of trials, a second light was illuminated after the initial GO cue directing the rat to inhibit their initial response to the first cue in favor of making a response in the direction of the second cue. ***b***, Illustration of GO (blue), STOP (red), STOP-error (dashed red) trial types. ***c***, ***d***, Percentage of correct and movement times for sequence effects: gG, go, go; sG, stop, go; sG, stop, go; sS, stop, stop. Percentage correct and movement times were averaged over sessions. Error bars represent ±SEM. ***e***, Movement time in the current trial as a function of trial success. GO, Blue; STOP, red. Movement times were averaged over sessions. Error bars represent ±SEM. ***f***, Electrode placements from seven rats that contributed neural data. ***g***, Zoomed-in schematic of electrode positions from ***f***. The sizes of circles represent the percentage of cells that significantly increased (black) or decreased (gray) firing during the response epoch (port exit to well entry) compared with baseline (1 s; Wilcoxon test, *p* < 0.0500).

Recordings were conducted in aluminum chambers 18 inches on each side with walls narrowing at the bottom of the arena to an area of 12 × 12 inches. On one wall, a central port was located above two adjacent fluid wells. Two lights were located above each fluid well, and house lights were located above the response panel. An illustration of the response panel is provided in Figure 1*a*. Task control was implemented via computer. Port entry and well entry times were monitored by disruption of photobeams.

The trial design is illustrated in Figure 1*a*. Each trial began with illumination of house lights that instructed the rat to nose poke into the central port. Nose poking initiated a 1000 ms precue delay period. At the end of this delay, a directional light to the left or right of the rat was flashed for 100 ms. If the rat exited the port at any time before offset of the directional cue light, the trial was aborted and house lights were extinguished. On 80% of trials (i.e., GO trials), presentation of the left or right light signaled the direction in which the rat could respond to obtain a sucrose reward in the corresponding fluid well below. On the remaining 20% of trials (i.e., STOP trials), the light opposite to the location of the originally cued direction turned on either at the same time as port exit or after a stop-signal delay (0–100 ms) and remained illuminated until the behavioral response was made. On STOP trials, rats were required to stop the movement signaled by the first light and respond in the direction of the second light. GO and STOP trials were randomly interleaved. On correct responding trials, rats were required to remain in the fluid well for a variable period between 800 and 1000 ms (prefluid delay) before reward delivery (10% sucrose solution). Error trials (incorrect direction) were immediately followed by the extinction of house lights and intertrial interval onset of 4 s. Trials were presented in a pseudorandom sequence such that left and right trials were presented in equal numbers (±1 over 250 trials).

##### Surgical procedures.

Rats were trained on the stop-change task for 1–2 months before undergoing electrode implantation surgery. All surgical procedures followed guidelines for aseptic technique. Electrodes were manufactured and implanted as in prior recording experiments (Bryden et al., 2011, 2012, 2016, 2019; Bryden and Roesch, 2015; Tennyson et al., 2018; Brockett et al., 2020). Rats were implanted unilaterally with a chronic electrode assembly that consisted of a drivable bundle of 10 FeNiCr wires (Stablohm 675, California Fine Wire) that were 25 µm in diameter targeting RN. Implants were counterbalanced across left and right hemispheres. Four animals were implanted at 5.2 mm posterior to bregma, 0.7 mm laterally, and 7.0 mm vertically down from the brain surface as in prior experiments (Roesch et al., 2007). The remaining three animals were implanted with a 5° angle pointed at the midline, with coordinates at 5.2 mm posterior to bregma, 1.4 mm laterally, and 7.5 mm vertically down from the brain surface. Immediately before implantation, wires were freshly cut with surgical scissors to extend 1 mm beyond the cannula and electroplated with platinum (H_2_PtCl_6_; Sigma-Aldrich) to an impedance of 300 kΩ. Immediately following surgery, rats were administered Rimadyl (5 mg/kg, s.c.), and the skin surrounding the surgical site was treated topically with a mixture of lidocaine and Neosporin. Rats also received injections of Rimadyl (5 mg/kg, s.c.), once daily for 2–3 d following surgery. Cephalexin (15 mg/kg, postoperative) was administered orally twice per day for 2 weeks postoperatively. After recording, rats were perfused, and their brains were removed and processed for histology as described previously (Bryden et al., 2011, 2012, 2016, 2019; Bryden and Roesch, 2015; Tennyson et al., 2018; Brockett et al., 2020). Briefly, once extracted, brains were postfixed for 48 h in 4% paraformaldehyde, cryoprotected in 30% sucrose, sectioned on a freezing microtome into 40 µm coronal sections, and Nissl stained for electrode placement verification and reconstruction using a light microscope (Bryden et al., 2011, 2012, 2016, 2019; Bryden and Roesch, 2015; Tennyson et al., 2018; Brockett et al., 2020).

##### Single-unit recordings.

Procedures for single-unit recordings in rats performing the stop-change task are the same as those described previously (Bryden et al., 2012, 2016, 2019; Bryden and Roesch, 2015; Tennyson et al., 2018; Brockett et al., 2020). Briefly, wires were screened for activity daily; if no activity was detected, the rat was removed and the electrode assembly was advanced 40 or 80 µm. If activity was detected, rats were allowed to perform the session, and the electrode was advanced at the end of the session. Neural activity was recorded using four identical Plexon Multichannel Acquisition Processor Systems. Signals from electrode wires were amplified 20× by an op-amp headstage located on the electrode array. Immediately outside the training chamber, signals were passed through a differential preamplifier (model PBX2/16sp-r-G50/16fp-G50, Plexon), where single-unit signals were amplified 50× and filtered at 150–9000 Hz. The single-unit signals were then sent to the Multichannel Acquisition Processor box, where they were further filtered at 250–8000 Hz, digitized at 40 kHz, and amplified at 1–32×. Waveforms (>2.5:1 signal-to-noise ratio) were extracted from active channels and recorded to disk by an associated workstation with event time stamps from the behavior computer.

##### Experimental design and statistical analysis.

Units were sorted via Offline Sorter (version 3.3; Plexon) using a template matching algorithm, and analyzed in Neuroexplorer (version 4.135; Plexon) and MATLAB (version 2017b; MathWorks). Brain activity was examined during the period between nose poke exit and well entry (response epoch). Activity presented in population histograms was normalized by dividing by the maximal firing rate of each neuron. Timing analyses were conducted comparing preferred and nonpreferred firing rates within a trial type using a sliding *t* test along 100 ms bins. Significant differences in firing between the preferred and nonpreferred directions are represented as colored bars. All statistical procedures were executed using raw firing rates (i.e., spikes per second).

Neurons were categorized as either “increasing” or “decreasing” by comparing the firing rate after port exit to fluid well entry (response epoch) to baseline firing (1 s before the start of central nose poke; Wilcoxon test, *p* < 0.05). Each neuron was further categorized by determining its preferred and nonpreferred direction (i.e., firing into and away from the response field of each neuron, respectively) by determining which direction produced the strongest response during the response epoch (i.e., nose poke exit to fluid well entry) averaged across all trial types. The direction that elicited the stronger firing was designated as the “preferred” direction (i.e., into the response field) and the opposite direction was designated “nonpreferred” direction (i.e., away from the response field). The directionality of firing was deemed significant if its firing rate differed from zero as indicated by a Wilcoxon signed-rank test.

For analysis of single units, we computed distributions of difference scores based on the raw firing rate (in spikes per second) for each neuron. To capture activity that differentiated based on previous trials, we examined firing rates on GO and STOP trials that followed either a GO or STOP trial. This analysis allows for the examination of sequence effects as well as comparisons between trials that were not preceded by a need to adapt behavior (i.e., when a STOP follows a GO) versus trials that were preceded by a need to adapt behavior (i.e., when a STOP follows a STOP). Abbreviations for these trials are differentiated by the trial type preceding it being denoted as lower case (i.e., “g” or “s” for GO or STOP). Distributions were deemed significant if they differed from either 0 or one another via Wilcoxon signed-rank and rank sum tests, respectively. For reference, the average number of trial types across sessions was as follows: GO, 140 ± 36; STOP, 34 ± 10; gG, 84 ± 25; sG, 13 ± 5; gS, 10 ± 5; sS, 15 ± 5 (values are presented as the mean number of trials per session ± SD).

For behavioral data, we analyzed two dependent variables, percentage correct and movement times. The percentage of correct scores was calculated by dividing the number of correct GO and STOP trials by the total number of trials. Movement time values were generated by calculating the time from center port exit to fluid well beam break. Similar calculations were made after filtering the data for sequence effects (i.e., when a GO preceded a STOP or GO or when a stop preceded either a STOP or GO). Planned *t* tests were conducted, where appropriate, to verify the directionality of interactions. Unless otherwise specified, all behavioral data (i.e., percentage correct or movement time data) were analyzed using a two-way ANOVA, where each datum is a session average so as to better reflect the presentation of the physiological data.

## Results

### Rats adjusted behavior within and across trials in response to STOP cues

Seven rats performed the stop-change task outlined in Figure 1*a*. In brief, rats began each trial by nose poking into the central port on illumination of houselights. After 1000 ms, one of two lights (left or right) was illuminated for 100 ms. On 80% of trials, rats responded in the direction of the light cue to obtain reward (GO trials). On 20% of trials, a second light cue was illuminated within 100 ms after the rat exited the central port. During these STOP-change trials, rats had to inhibit their initial movement in the direction of the first light and redirect their movement in the direction of the second light to obtain reward. For all trials, reward was delivered 800–1000 ms after entering the fluid well. In total, there were four possible trial types, as follows: go-left, go-right, stop-left-go-right, and stop-right-go-left (Fig. 1*b*). Percentage correct and movement times (i.e., time from nose port exit till well entry) were calculated across session averages to better match the presentation of the physiological data.

As reported previously (Bryden et al., 2012, 2016, 2019; Bryden and Roesch, 2015; Tennyson et al., 2018; Brockett et al., 2020), rats exhibited decreased accuracy on STOP relative to GO trials (*t* test: *t*_(1052)_ = 14.848, *p* = 2.2 × 10^−16^). Rats were slower on STOP correct compared with GO correct trials, but were faster on STOP error compared with STOP correct trials (ANOVA; interaction of trial type × performance: *F*_(1,2104)_ = 27.982; *p* = 1.3 × 10^−7^; GO correct vs STOP correct: *t* test: *t*_(526)_ = −6.4504, *p* = 2.5 × 10^−10^); STOP correct vs STOP error: *t* test, *t*_(524)_ = 2.2939, *p* = 0.0222; Fig. 1*e*). Increased difficulty and fast errors both suggest that rats were planning fast automatic responses to the first cue, which then had to be inhibited and redirected.

To determine whether rats adjusted behavior across trials we examined whether trial history altered accuracy (Fig. 1*c*) or movement times (Fig. 1*d*) by performing an ANOVA with current and previous trial type as factors. Consistent with the analysis above, rats were less accurate on STOP versus GO trials (ANOVA; main effect of current trial: *F*_(1,2104)_ = 250.43, *p* = 2.0 × 10^−16^; Fig. 1*c*) and slower (ANOVA; main effect of current trial: *F*_(1,2104)_ = 145.29, *p* = 2.0 × 10^−16^; Fig. 1*d*). Accuracy was also significantly affected by previous trial type or trial history (ANOVA; main effect of previous trial: *F*_(1,2104)_ = 45.670, *p* = 1.8 × 10^−11^). Moreover, we observed an interaction between current trial performance and previous trial (ANOVA; interaction of current trial × previous trial: *F*_(1,2104)_ = 76.410, *p* = 2.0 × 10^−16^), suggesting that while rats were worse at STOP trials overall, accuracy was modulated by trial history [i.e., rats were more accurate when a STOP trial preceded another STOP trial (i.e., sS) than when a GO trial preceded a STOP trial (i.e., gS; *t*_(526)_ = −6.5679, *p* = 1.2 × 10^−10^; Fig. 1*c*].

Collectively, these findings suggest that rats performed worse on STOP trials when compared with GO trials and that rats modulated their responding across trials. Thus, rats demonstrate that they are able to adapt their ability to respond to two competing action plans based on previous experience.

### Directional response signals in RN were amplified during STOP trials

**Figure 2.**
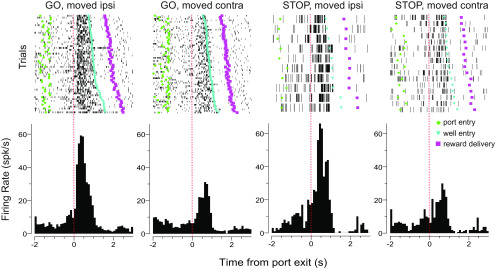
Raster plots showing an example of a directionally selective increasing cell. From left to right figures show the following: GO trials where the rat moved in the direction ipsilateral to the electrode location; GO trials where rats moved in the contralateral direction; STOP trials where the rat inhibited the contralateral movement (signaled by the first light) and moved in the ipsilateral direction (signaled by the second light); and STOP trials where the rat inhibited the ipsilateral movement and moved in the contralateral direction. Sessions are sorted by movement speed. Plots are aligned to port exit. Green diamonds reflect port entry, blue upside down triangles represent well entry, purple squares represent reward delivery, and each tick mark represents an action potential.

**Figure 3.**
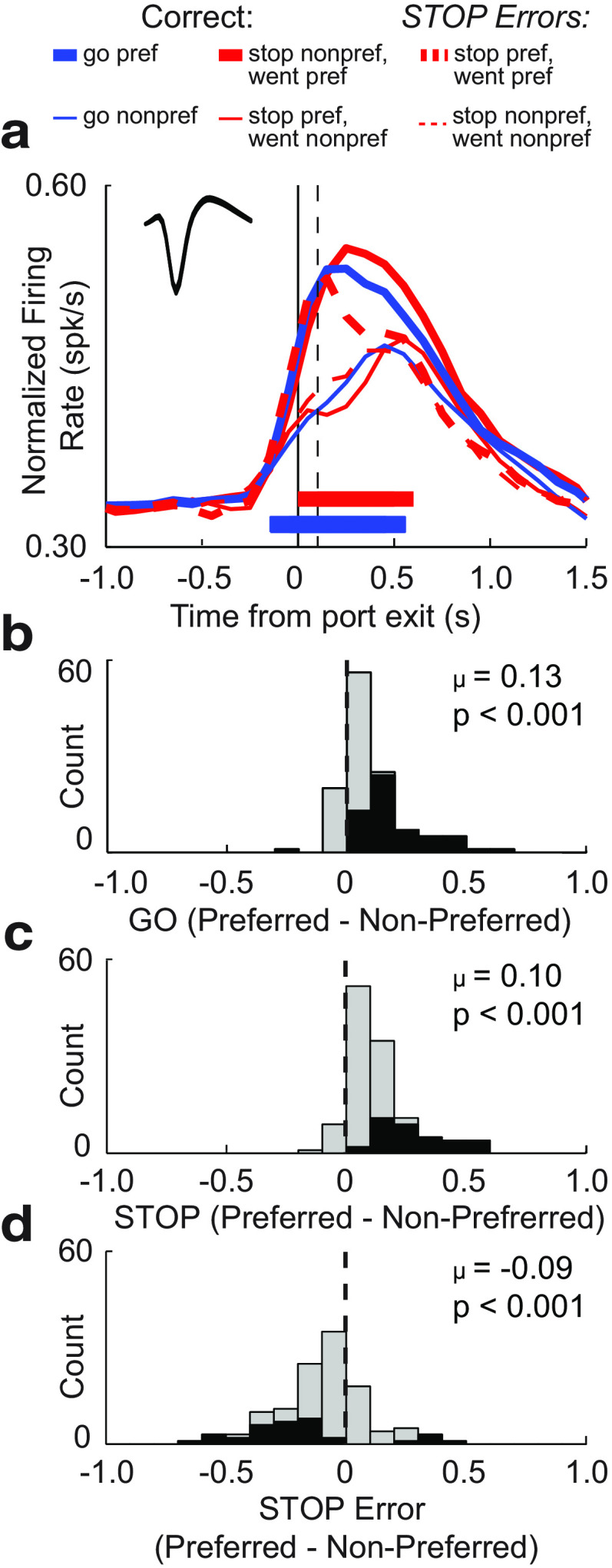
RN firing was higher for STOP trials when aligned to port exit for increasing cells (*n* = 121). ***a***, Average population histogram for all trial types aligned to time of cue onset. Red, STOP; Blue, GO; solid, correct; dashed, error; thick, preferred direction; thin, nonpreferred direction. Preferred direction was determined by the direction that elicited the stronger response average across correct trial types during the response epoch (port exit to well entry) for each neuron. Inset shows the average waveform shape (maximum to maximum). ***b–d***, Distribution of directional indices (preferred − nonpreferred/preferred + nonpreferred) computed during the response epoch for GO (***b***), STOP (***c***), and STOP-error (***d***) trials (Wilcoxon test, μ = mean). Black bars indicate individual cells that exhibited significant differences between preferred and nonpreferred directional responses (Wilcoxon test, *p* < 0.05).

We recorded 527 RN neurons from seven rats performing the stop-change task. One hundred twenty-one of these neurons (23%) exhibited increases in firing during the response epoch (central port exit to well entry) relative to baseline (1 s starting 2 s before nose poke; Wilcoxon test, *p* < 0.05; Fig. 2). For increasing cells, we observed a significant difference in the number of cells firing more strongly for ipsilateral (*n* = 42) versus contralateral (*n* = 15) movements relative to the recording electrode (χ^2^ = 12.690, *p* = 0.0003). Figure 3*a* illustrates average firing aligned to port exit for GO (blue) and STOP-change (red) trials for movements made into (preferred direction; thick) and away from (nonpreferred direction; thin) the response field of each neuron (see Materials and Methods; preferred direction was defined as the direction that elicited the highest response average over trial types; Bryden et al., 2011, 2012, 2016, 2019; Bryden and Roesch, 2015; Tennyson et al., 2018; Brockett et al., 2020).

For both GO (blue) and STOP (red) trials, average RN firing increased on illumination of the imperative light (i.e., first light on GO trials and second light on STOP trials), reflecting the direction of the upcoming movement (Fig. 3*a*). Directional selectivity on STOP trials emerged within 100 ms after port exit (difference between thick and thin lines; *t* tests across 100 ms bins: *p* values < 0.0100), before the stop-change reaction time (SCRT; vertical dashed line; STOP minus GO movement time; time necessary to stop and redirect), suggesting that firing in RN can contribute to the correct motor output.

During STOP trials we also saw that the directional signal was amplified after the SCRT. That is, firing was higher (thick red vs thick blue) and lower (thin red vs thin blue) for movements made into and away from the response field, respectively. As a result, how well the RN population discriminated between left and right movements (i.e., the strength of the directional signal) was stronger under STOP compared with GO trials. This boost in the directional signal occurred after the decision to stop and redirect behavior, during the period of time when the rat was completing the behavioral response.

To quantify this effect, we computed directional indices for GO and STOP trials (preferred − nonpreferred/preferred + nonpreferred) using the average firing during the response epoch for each neuron (Figure 3*b–d*). Consistent with the population firing observed in Figure 3*a*, the distributions of directional indices for both GO and STOP trials were shifted in the positive direction [Wilcoxon test; GO, *p* < 0.0010; µ = 0.10 (Fig. 3*b*); Wilcoxon; STOP, *p* < 0.0010; µ = 0.13 (Fig. 3*c*)], and was significantly stronger for STOP compared with GO trials (Wilcoxon test; *z* = 2.01; *p* = 0.0440). Thus, the majority of increasing-type RN cells exhibited stronger differences in firing between movements made into and away from the response field on STOP compared with GO trials.

### Activity on STOP trials reflected the errant motor response

The behavior of rats during the performance of errant STOP trials suggests that they were rapidly responding to the first cue light. Consistent with this behavioral observation, activity on STOP-error trials rapidly reflected the direction associated with the first cue, not the second cue, thus tracking the direction of the errant movement. This is best illustrated by comparing thick blue to thick red dashed lines in Figure 3*a*. Thick red dashed trials are STOP trials where the first cue was in the response field of the neuron, but the second cue was opposite the response field. On these trials, activity rapidly increased similar to GO trials where the first cue is in the same direction (thick blue). Interestingly, the immediate increase in firing before the SCRT rapidly declined after the SCRT. This suggests that even after firing crossed the decision threshold to move (i.e., point of no return), RN attempted to shut down firing, albeit without success. Consistent with these observations, the number of significant neurons that incorrectly encoded the wrong direction on error trials significantly outnumbered the count of neurons that encoded the correct direction (Fig. 3*d*, black bars 29 vs 5; χ^2^ = 16.800; *p* =3.8 × 10^−5^). Further, the distribution of directional indices on STOP-error trials was significantly shifted in the negative direction (Wilcoxon test; *p* < 0.0010; µ = −0.09; Fig. 3*d*), reflecting the direction of the errant movement as opposed to the location of the second cue light.

### GO directional signals emerge more slowly after STOP trials

**Figure 4.**
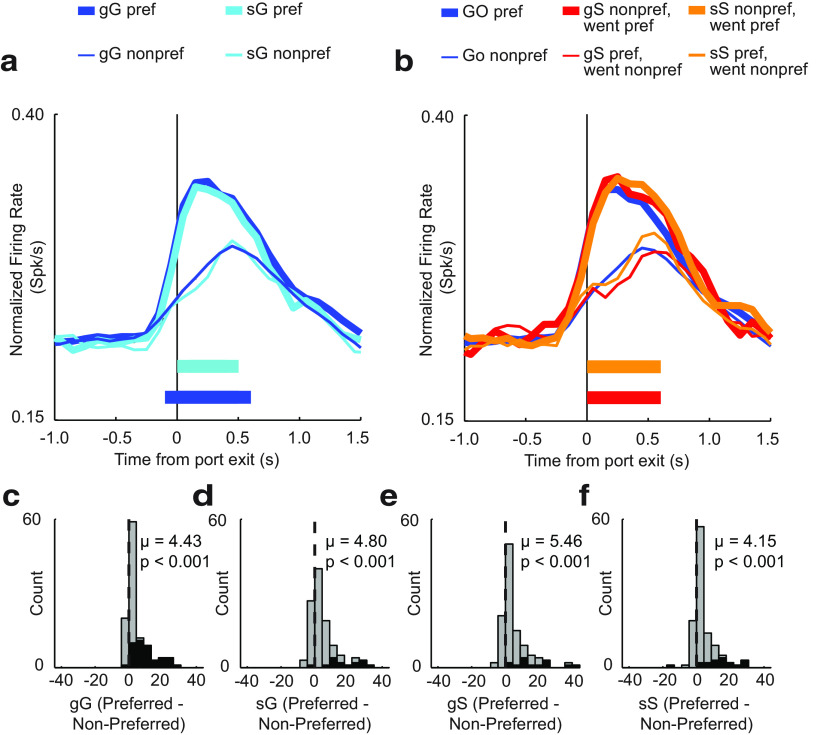
Effects of trial sequence on RN firing for increasing cells. ***a***, Population histogram aligned to port exit for trials followed by a GO trial: gG (blue); sG (teal). Line thickness indicates direction, preferred (thick) or nonpreferred (thin). ***b***, Population histograms comparing average GO trials (preferred: thick, blue; nonpreferred: thin, blue) to gS trials (preferred: thick, red; nonpreferred: thin, red) and sS trials (preferred: thick, orange; nonpreferred: thin, orange). Aligned to time of port exit. ***c–f***, Distributions of directional indices (preferred − nonpreferred) for gG (***c***), sG (***d***), gS (***e***), and sS (***f***) or all trial types (Wilcoxon test, μ = mean). Black bars indicate individual neurons that exhibited a significant shift (Wilcoxon test, *p* < 0.05).

The underlying premise behind stop-tasks is the notion that participants build up a habitual and automatic tendency toward rapidly responding to the first cue. This priming of the motor system makes it difficult to adjust behavior when a STOP cue is presented. However, once the chain of GO trials is broken by a STOP trial, participants become more cautious, exhibiting a higher probability of success on subsequent STOP trials. One neural mechanism by which this might occur is to proactively disengage motor structures that are driving the initial GO response, giving more time for signals that inhibit and redirect movement in the opposite direction to emerge. Interestingly, this is what we observed for increasing-type cells recorded in RN, as illustrated in Figure 4*a*.

In Figure 4*a*, on gG trials (blue; GO followed by GO), significant directional selectivity [i.e., difference between directions (thick vs thin)] emerged within the 100 ms bin preceding port exit, whereas on sG trials (teal; STOP followed by GO trial), the directional selectivity did not emerge significantly until the 100 ms bin after port exit (Fig. 4*a*; 100 ms bins; *t* test, *p* < 0.0100). Thus, after STOP trials, RN did not signal the direction of the first cue light until after initiation of the movement.

Next, we asked whether the overall strength of the directional signal was weaker on gG compared with sG trials. One could imagine that in addition to delaying the signaling of the first cue light, decreasing the strength of the neural response driving the response could mechanistically promote better response inhibition. To address this hypothesis, we computed directional indices (preferred − nonpreferred) during the response epoch for all trial sequences (gG, sG, gS, and sS; Fig. 4*c–f*). Distributions for all trial sequences were significantly shifted above 0 for all trial types [Wilcoxon tests; gG: *p* < 0.0010; µ = 4.43 (Fig. 4*c*); sG trials: *p* < 0.0010; µ = 4.80 (Fig. 4*d*); gS: *p* < 0.0010; µ = 5.46 (Fig. 4*e*); sS: *p* < 0.0010; µ = 4.15 (Fig. 4*f*)]; however, there were no significant differences between gG and sG (Wilcoxon test; *z* = 0.14; *p* = 0.8890) or between sS and gS directional index distributions (Wilcoxon test; *z* = 0.83; *p* = 0.4000). Thus, it appears that the only role that increasing-type cells may contribute to trial-by-trial adjustments of behavior is to initiate movement signals to the first cue light more slowly after STOP trials.

### Increasing-type RN neurons were negatively correlated with movement time

The results above strongly suggest that increases in firing promote behavior toward the response field of RN neurons. To better understand the relationship between firing and movement speed, we performed two different analyses, one at the population level and the other within single neurons.

**Figure 5.**
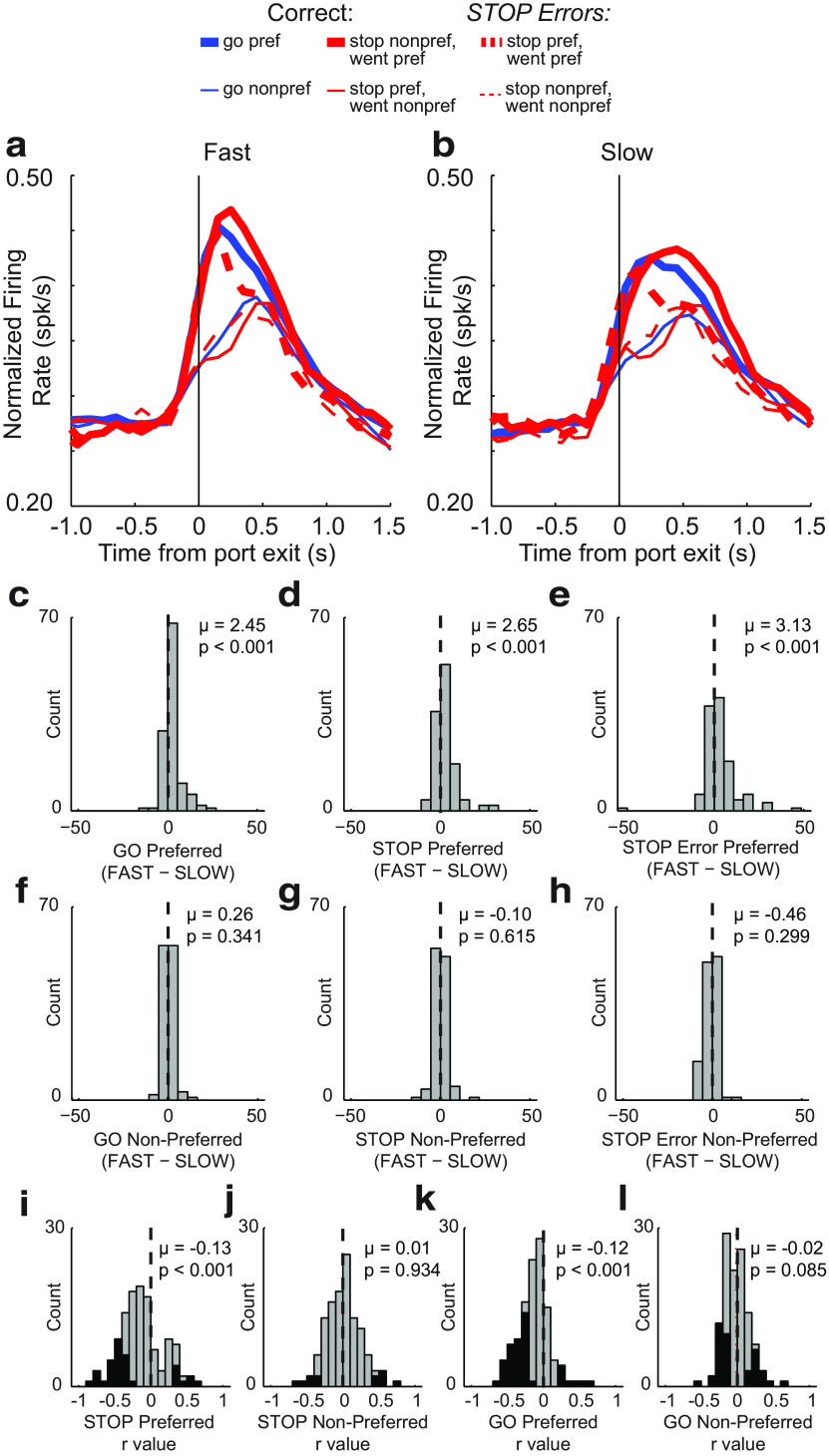
Effects of movement time on RN firing on increasing cells. ***a***, ***b***, Average population histogram for Fast (***a***) and Slow (***b***) trials. Fast and Slow were determined by taking the median split within each recording session. Trial types are distinguished by color, as follows: GO, blue; STOP, red; STOP-error, dashed red. Direction is indicated by line thickness, as follows: preferred, thick; nonpreferred, thin). ***c–h***, Distribution of speed indices comparing firing during Fast to Slow trials (Fast −Slow) for GO preferred (***c***) and nonpreferred (***f***); STOP preferred (***d***) and nonpreferred (***g***); and STOP error preferred (***e***) and nonpreferred (***h***; Wilcoxon test: *p* < 0.05; μ represents the mean). ***i–l***, Distribution of *r* values depicting correlation between firing rate during the response epoch and movement time for STOP preferred (***i***) and nonpreferred (***j***) and GO preferred (***k***) and nonpreferred (***l***) directions (Wilcoxon test: μ = mean). Black bars indicate individual neurons that exhibited significant within-session correlations between firing rate and movement time (*p* < 0.05).

In the first analysis, we replotted the average population histogram, splitting trials into fast and slow movement times (median split) within each session. We then calculated indices to compare firing rates on fast and slow trials for each trial type (GO, STOP, STOP-error) in both directions (preferred and nonpreferred). Figure 5, *a* and *b*, shows the average RN activity plotted for fast (Fig. 5*a*) and slow (Fig. 5*b*) trials. As in the grand average population histogram plot (Fig. 3*a*), directional signals were amplified on STOP trials. Here, by breaking down trials into fast and slow, we are able to visualize a close correspondence to motor output in that phasic increases in firing were stronger and more rapid during faster trials (Fig. 4, compare *a*, *b*). To quantify this effect, we computed a new index comparing firing on fast versus slow trials (fast − slow) during the response epoch for all trial types, independently for preferred and nonpreferred movement directions. For GO, STOP, and STOP-error trials, all distributions were significantly shifted above 0 [Wilcoxon tests; GO: *p* < 0.0010; µ = 2.45 (Fig. 5*c*); STOP: *p* < 0.0010; µ = 2.65 (Fig. 5*d*); STOP error: *p* < 0.0010; µ = 3.13 (Fig. 5*e*)] for movements made in the preferred direction, but not in the nonpreferred direction [Wilcoxon tests; GO: *p* = 0.3410; µ = 0.26 (Fig. 5*f*); STOP: *p* = 0.6150; µ = −0.10 (Fig. 5*g*); STOP error: *p* = 0.2990; µ = −0.46 (Fig. 5*h*)], indicating that stronger firing was associated with faster responding in the preferred direction across all trial types.

These results suggest that increases in firing across the population of RN neurons drives movement. To determine whether this was also evident for the firing of single neurons, we performed a regression analysis to determine how many neurons exhibited firing during the response epoch that correlated with movement time. The distribution of *r* values for GO and STOP trials in the preferred and nonpreferred directions are plotted in Figure 5*i–l*. For both GO and STOP trials, the distribution of *r* values was significantly shifted in the negative direction only for movements made in the preferred direction [Wilcoxon tests; STOP: *p* < 0.0010; µ = −0.13 (Fig. 4*i*); GO: *p* < 0.0010; µ = −0.12 (Fig. 5*k*)] and the counts of neurons that showed a significant negative correlation between firing rate and movement time significantly outnumbered those showing a positive correlation for STOP and GO trials made in the preferred direction (Fig. 5*i*,*k*, black bars; STOP: 38 vs 8; χ^2^ = 19.400; *p* < 0.0010; GO: 25 vs 8; χ^2^ = 8.6500; *p* = 0.0030).

### RN neurons that decrease firing were less inhibited on STOP trials

**Figure 6.**
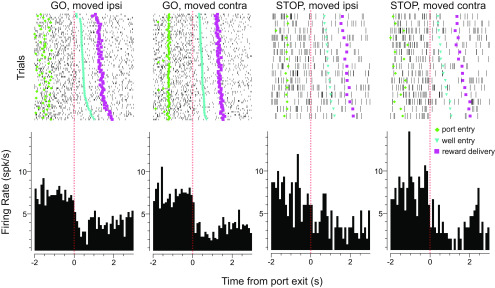
Raster plots showing an example of a directionally selective decreasing cell. From left to right: GO trials where the rat moved in the direction ipsilateral to the electrode location; GO trials where rats moved in the contralateral direction; STOP trials where the rat inhibited the contralateral movement signaled by the first light and moved in the ipsilateral direction (signaled by the second light); and STOP trials where the rat inhibited the ipsilateral movement and moved in the contralateral direction. Sessions are sorted by movement speed. Plots are aligned to port exit. Green diamonds reflect port entry, blue upside down triangles represent well entry, purple squares represent reward delivery, and each tick mark represents an action potential.

**Figure 7.**
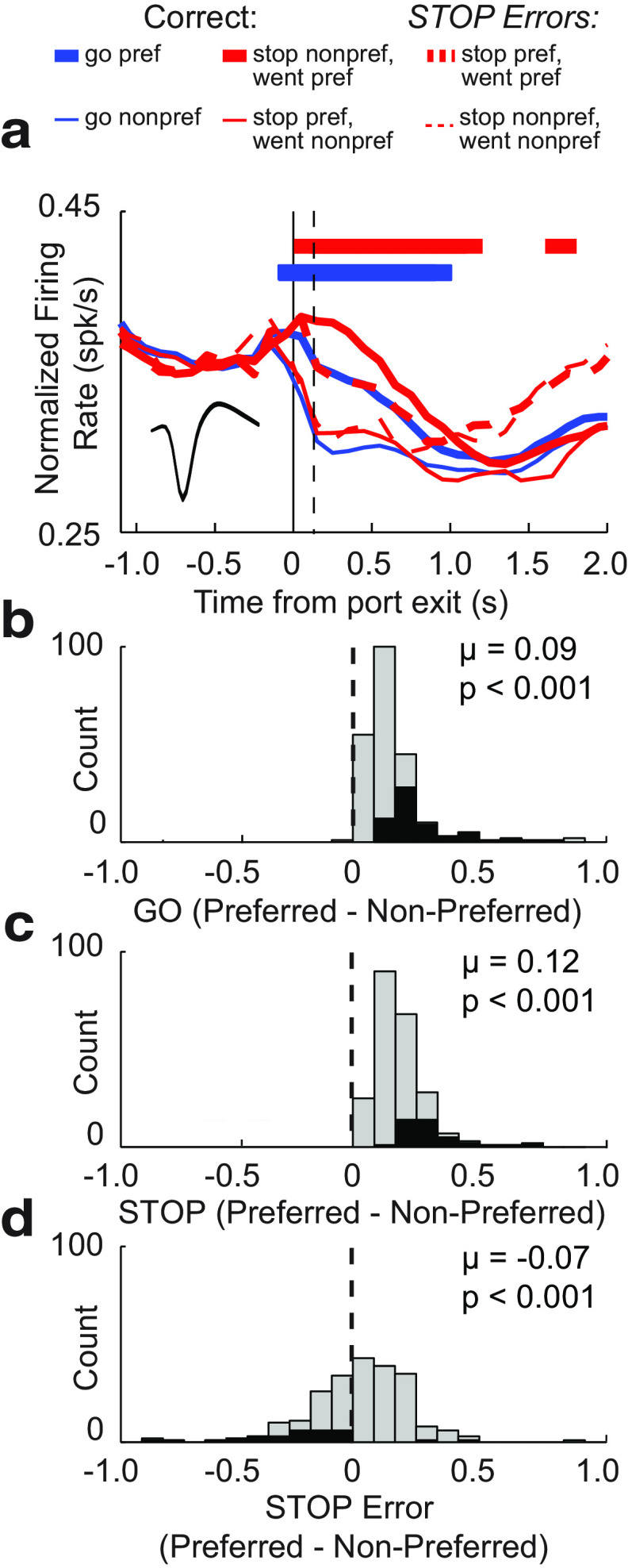
RN firing was higher for STOP trials when aligned to port exit for decreasing cells (*n* = 229). ***a***, Average population histogram for all trial types aligned to time of cue onset. Red, STOP; Blue, GO; solid, correct; dashed, error; thick, preferred direction; thin, nonpreferred direction. Preferred direction determined by the direction that elicited the stronger response average across correct trial types during the response epoch (port exit to well entry) for each neuron. Inset shows average waveform shape (maximum to maximum). ***b–d***, Distribution of directional indices (preferred − nonpreferred/preferred + nonpreferred) computed during the response epoch for GO (***b***), STOP (***c***), and STOP-error (***d***) trials (Wilcoxon test, μ = mean). Black bars indicate individual cells that exhibited significant differences between preferred and nonpreferred directional responses (Wilcoxon test, *p* < 0.05).

Above we examined neurons that increased firing during the response epoch, here we repeat the identical analysis but for cells that significantly decreased firing during the response epoch [*n* = 229 (43%); Wilcoxon test; *p* < 0.05; Fig. 6]. Increasing and decreasing cell waveforms (Figs. 3*a*, 7*a*, insets; increasing = 407 µs; decreasing = 421 µs) and baseline firing rates (increasing = 11.9 spikes/s; decreasing = 10.5 spikes/s) did not differ significantly from each other (duration: *t* test, *t*_(348)_ = 1.9000; *p* = 0.3200; baseline: *t* test, *t*_(348)_ = 1.9000; *p* = 0.2900). However, there was a significant difference in the frequency of cells that fired differently for ipsilateral versus contralateral movements (χ^2^ = 9.330, *p* = 0.0020). Unlike increasing-type cells, for decreasing-type cells, the counts of neurons that fired significantly more for ipsilateral movement (*n* = 27) did not significantly outnumber those that fired significantly more for contralateral (*n* = 34) movement (χ^2^ = 0.7800, *p* = 0.3700). However, despite this difference, much like increasing-type cells, directional signals for decreasing-type cells significantly emerged within 100 ms after illumination of the imperative cue on correct trials and were stronger on STOP trials compared with GO trials; however, the increase in directional signal solely arose from higher firing in the preferred direction [thick vs thick blue; 30 cells (13%) showed significantly stronger firing on STOP trials compared with GO trials in the preferred direction].

Distributions of directional indices for each trial type are plotted in Figure 7*b–d* for decreasing-type cells. Indices were shifted significantly more strongly above 0 on STOP trials compared with GO trials (Wilcoxon tests; GO, *p* < 0.0010; µ = 0.09 (Fig. 7*b*); STOP, *p* < 0.0010; µ = 0.12 (Fig. 7*c*); GO vs STOP: *z* = 3.72; *p* < 0.0010) and was shifted below 0 when STOP errors were made (Wilcoxon test; *p* < 0.0010; µ = −0.07; Fig. 7*e*).

### Directional signals on GO and STOP trials were strengthened when the previous trial was of the same trial type

**Figure 8.**
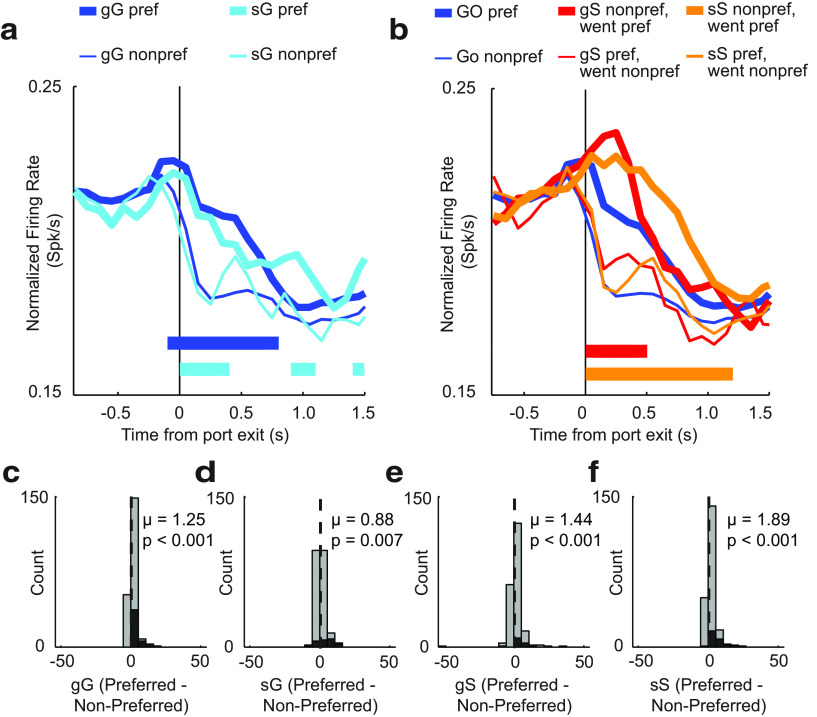
Effects of trial sequence on RN firing for decreasing cells. ***a***, Population histogram aligned to port exit for trials followed by a GO trial: gG, blue; sG, teal. Line thickness indicates direction preference: preferred, thick; or nonpreferred, thin. ***b***, Population histograms comparing average GO trials (preferred: thick, blue; nonpreferred: thin, blue) to gS (preferred: thick, red; nonpreferred: thin, red) and sS trials (preferred: thick, orange; nonpreferred: thin, orange). Activity is aligned to time of port exit. ***c–f***, Distributions of directional indices (preferred − nonpreferred) for gG (***c***), sG (***d***), gS (***e***), and sS (***f***) or all trial types (Wilcoxon test, μ = mean). Black bars indicate individual neurons that exhibited a significant shift (Wilcoxon test, *p* <0.05).

As described above, rats were better on trials when the previous trial was of the same trial type. That is, rats are better on sS and gG trials compared with gS and sG trials, respectively. As before, one neural mechanism that might govern this behavior is to slow and weaken the development of directional motor signals to the first cue light, thus allowing signals related to inhibition and redirection time to take effect (i.e., win the race). On GO trials, directional signals emerged earlier (Fig. 8*a*, blue vs teal ticks), and the distribution of directional indices were significantly more positive on gG trials compared with sG trials [Wilcoxon tests; Fig. 8*c*: gG, *p* < 0.0010; µ = 1.25; Fig. 8*d*: sG, *p* = 0.0060; µ = 0.88; vs sG,*z* = 2.49; *p* = 0.0130]. Thus, directional signals were attenuated and developed more slowly on GO trials that occurred after STOP trials, similar to what we saw before with increasing-type cells.

On STOP trials, we found that the directional indices were also shifted significantly more positive on sS trials compared with gS trials [Wilcoxon tests; gS: *p* < 0.0010; µ = 1.44 (Fig. 8*e*); sS: *p* < 0.0010; µ = 1.89 (Fig. 8*f*)]; however, this did not achieve significance; Wilcoxon test, gS vs sS: *z* = 1.16; *p* = 0.2500). Interestingly, however, directional signals persisted longer on sS trials compared with gS trials (Fig. 8*b*, red vs orange tick marks; *t* tests across 100 ms bins, *p* values < 0.0100).

### Decreasing-type RN neurons were positively correlated with movement time

**Figure 9.**
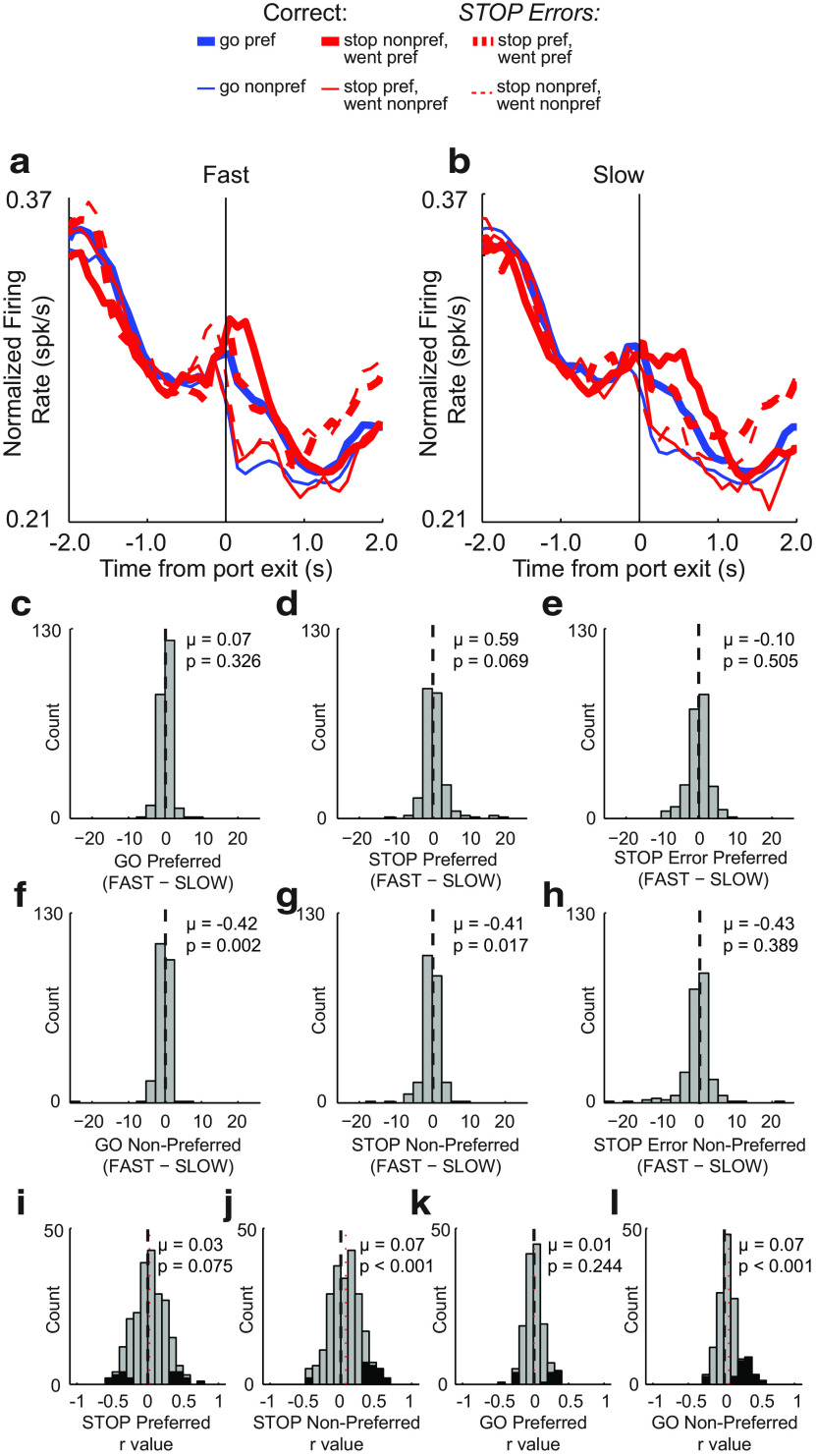
Effects of movement time on RN firing on decreasing cells. ***a***, ***b***, Average population histogram for Fast (***a***) and Slow (***b***) trials. Fast and Slow were determined by taking the median split within each recording session. Trial types are distinguished by color: GO, blue; STOP, red; STOP-error, dashed red. Direction is indicated by line thickness: preferred, thick; nonpreferred, thin. ***c–h***, Distribution of speed indices comparing firing during Fast to Slow trials (Fast − Slow) for GO preferred (***c***) and nonpreferred (***f***); STOP preferred (***d***) and nonpreferred (***g***); and STOP error preferred (***e***) and nonpreferred (***h***; Wilcoxon test, μ = mean). ***i–l***, Distribution of *r* values depicting the correlation between firing rate during the response epoch and movement time for STOP preferred (***i***) and nonpreferred (***j***) and GO preferred (***k***) and nonpreferred (***l***) directions (Wilcoxon test, μ = mean). Black bars indicated individual neurons that exhibited a significant within-session correlations between firing rate and movement time (*p* < 0.05).

To determine whether RN activity of decreasing-type cells was modulated by the speed with which rats responded, average population histograms were again split into fast and slow trials based on movement times, and speed indices (fast-slow) were computed for each neuron, as described above. For these neurons, distributions were not significantly shifted from 0 for increases in firing [Wilcoxon tests; preferred direction: GO, *p* = 0.3260; µ = 0.07 (Fig. 9*c*); STOP, *p* = 0.0700; µ = 0.59 (Fig. 9*d*)]; however, the distributions for movements associated with decreased firing were significantly shifted in the negative direction for both correct GO and STOP trials (Wilcoxon tests; GO, *p* = 0.0020; µ = −0.42 (Fig. 9*f*); STOP, *p* = 0.0170; µ = −0.41 (Fig. 9*g*)].

These results suggest that decreases—not increases—in firing promote movement for decreasing-type cells. To determine whether this was also true within single units, we performed a regression analysis to determine in how many neurons firing during the response epoch was correlated with movement time (Fig. 9*i–l*). For both GO and STOP trials, the distribution of *r* values was significantly shifted in the positive direction only for movements that elicited decreases in firing [Wilcoxon tests; STOP: *p* < 0.0010. µ = 0.07 (Fig. 9*k*); GO: *p < 0.0010*; µ = 0.07 (Fig. 9*l*)]. Further, at the single-neuron level, the counts of individual neurons that showed a significant positive correlation between firing rate and movement time significantly outnumbered those showing a negative correlation (Figs. 9*j*,*l*, black bars; GO: 2 vs 20; χ^2^ = 14.300, *p* < 0.0010; STOP: 4 vs 37; χ^2^ = 26.400, *p* < 0.0010).

## Discussion

In this study, we recorded from RN neurons in rats performing a stop-change task. We found that these motor signals were modulated during adjustments in behavior that occurred within and across trials. Within-trial adjustments in behavior in response to the STOP cue were accompanied by amplified directional signals. Across trial adjustments in behavior, which occurred after experiencing a STOP trial, were accompanied by a slower and weaker representation of the directional signal on GO trials, consistent with changes in behavior that occur in these trial types.

Overall, our results are consistent with previous work examining single-neuron activity in animals performing goal-directed movement related to reaching and grasping (Ghez and Kubota, 1977; Amalric et al., 1983; Cheney et al., 1988; Jarratt and Hyland, 1999; van Kan and McCurdy, 2001; Van Kan and McCurdy, 2002; Pacheco-Calderón et al., 2012; Herter et al., 2015). One way that RN appears to contribute to task performance is in boosting directional signals on STOP trials. Immediately on port exit, neurons in RN reflected the direction of the response. The rapid onset of directional signal on STOP trials suggests the priming of both actions, which is then increased, relative to GO trials, after presentation of the STOP cue, but only when the rat was successful at inhibiting its initial motor plan. On error trials, it appears that the RN tries to shut down the already initiated movement, but is unsuccessful. For increasing cells, the boost in directional signals occurs after the SCRT in both the preferred and nonpreferred direction, suggesting that these cells contribute more to the modulation of the ongoing motor plan on STOP trials as opposed to the neural signals that are signaling inhibition and redirection. Since the firing of these neurons exhibits a strong negative correlation with movement speed in the preferred direction, we propose that the primary contribution of these neurons during STOP trials is to drive behavior toward the response field of each neuron so that the redirected movement can be completed.

For decreasing-type cells, the relationship between firing rate and movement time is the opposite. That is, increases and decreases in firing associated with a slowing and speeding up of motor output, respectively. This suggests that the tonic firing of decreasing neurons inhibits behavior and that decreases in tonic firing during trial events (nose poke, port exit, fluid well entry) remove this inhibition, allowing movements to occur. Notably, increases in firing observed in STOP trials on port exit are observed before the SCRT, suggesting that they likely contribute to inhibition of the motor response signaled by the first cue light, while allowing appropriate actions to made in the opposite direction.

RN also appears to contribute to adjustments in behavior that occur trial-to-trial that are induced by the identity of the preceding trial type. After successful completion of a GO trial, rats build an automatic tendency to quickly respond to the first light to obtain reward; thus, if the current trial is a GO trial, this leads to a fast successful response. However, if the current trial is a STOP trial, the likelihood of an error being made is higher, and if successful inhibition does occur, it takes much longer (compared with sS trials). After the completion of a STOP trial, rats tend to respond more slowly to the first cue light and are better prepared to respond to the second cue light. Thus, after STOP trials, rats tend to be better at STOP trials (i.e., conflict adaption), but are worse at GO trials. Remarkably, RN appears to contribute to these processes. For increasing cells, which drive behavior (i.e., negative correlation between firing and movement time), directional selectivity emerged earlier and persisted longer on GO trials that followed GO trials (gG); however, the strength and emergence of the directional signal was not different between sS and gS trials. Thus, if increasing-type cells were to play a role in altering behavior after GO and STOP trials, it would be to drive behavior toward the first cue light more or less rapidly, respectively. For decreasing-type cells, the strength of the directional signal was stronger on gG trials compared with sG trials. Thus, the strength—not just the timing—of the directional signal was modulated by trial history.

Historically, RN has been studied for its contributions to motor processes (Cheney et al., 1991; Holstege, 1991; Houk, 1991). In primates, RN is important for the initiation of fine motor control of distal processes, and lesion/inactivation studies produce mild to moderate deficits in these abilities (Lawrence and Kuypers, 1968; Larsen and Yumiya, 1980; Kennedy et al., 1986). In cats, RN lesions produce deficits in the initiation of motor responses along with abnormal gait and other locomotor difficulties (Ingram and Ranson, 1932; Evans and Ingram, 1939; Smith, 1970; Orlovsky, 1972; Amalric et al., 1983; Gibson et al., 1985; Batson and Amassian, 1986; Amassian and Batson, 1988; Arshavsky et al., 1988; Martin and Ghez, 1988; Schmied et al., 1988; Levesque and Fabre-Thorpe, 1990). In rodents, unilateral lesions to RN also produce motor initiation and gait-related issues for movements in the direction contralateral to the lesion (Muir and Whishaw, 2000) as well as deficits in fine motor skills (Whishaw et al., 1990; Rizzi et al., 2019), although recent work in mice suggests that deficits in motor output are restricted to fine motor control and not gross locomotion (Rizzi et al., 2019).

Despite considerable evidence implicating RN in the initiation and control of motor responses, relatively few studies have investigated RN, particularly the parvocellular portion of RN (RNp), in cognitive functions (Thompson et al., 1967; McNew, 1968; Habas and Cabanis, 2006, 2007; Nioche et al., 2009). Using resting-state diffusion tensor imaging methods, work in humans has suggested that RN receives numerous direct connections from the cortex, including areas such as prefrontal cortex, and that RN activity does not correlate with activity in motor areas, instead correlating most strongly with the activity of brain areas associated with salience and executive control networks (Nioche et al., 2009). In principle, the idea that RN may be important for cognition is supported by visual memory studies in rats that found bilateral lesions of RN impaired retention of visual stimuli in a visual discrimination task (Thompson et al., 1967; McNew, 1968). Moreover, lesion studies in rabbits looking at eye-blink conditioning have shown that damage to RN disrupts the expression of the conditioned response, without disrupting memory of the response, which is held in the cerebellum (Rosenfield and Moore, 1983; Krupa et al., 1993). In this light, these findings may suggest that in our task, lesions to RN may disrupt behavioral performance via a failure to integrating cognitive control signals from frontal regions rather than a deficit in pure motor output.

Across evolution, the size of the RNp has steadily grown (Holstege, 1991; Nioche et al., 2009; Aghoghovwia and Oorschot, 2016). Compared with felines, a major model system in RN function studies, rodents are estimated to have on average ∼1400 more parvocellular neurons than magnocellular neurons in RN (Aghoghovwia and Oorschot, 2016). Although speculative, rodents may represent a branching point in the remodeling of the functional output of RN, and detailed mapping studies of rodent RN along with cross-species comparison studies may be informative in shaping thoughts on RN function moving forward.

One limitation of our present findings is that, although our electrode coordinates primarily targeted RNp, it is difficult to rule out some contribution of the magnocellular division. Electrode tracts followed our intended coordinates (Fig. 1*f*,*g*), and, to our knowledge, there is limited evidence suggesting that these cell populations can be separated on the basis of electrophysiological properties. Despite this limitation, one possibility for linking rapid changes in cognitive outputs with downstream motor outputs may involve RNp, which receives both cortical and cerebellar projections (Swenson and Castro, 1983; Onodera, 1984; Onodera and Hicks, 2009). The RNp sends projections to the inferior olive, the point of origin for the climbing fibers that enwrap Purkinje neurons in the cerebellum. Climbing fibers have strong modulatory effects on Purkinje neurons (Loewenstein et al., 2005; Schonewille et al., 2006; Yartsev et al., 2009; Forrest, 2014) and seem well positioned to relay information about sudden changes in either motor plan or task contingencies. When viewed from the perspective of the stop-change task, the RNp may act as a point of integration on STOP-trials, which require both the rapid detection of conflict as well as the near-immediate reshaping of a motor plan or ongoing motor sequence.

Collectively, these results suggest that RN neurons contribute to adjustments in behavior during response inhibition and cognitive control. Modulation of firing in RN likely reflects direct and indirect inputs from cortex and striatum that are setting the tone for responding, as well as feedback from motor structures signaling the need to boost directional signals when there is competition between opposite motor acts.
